# Friend or Foe? Defining the Role of Glutamate in Aging and Alzheimer’s Disease

**DOI:** 10.3389/fragi.2022.929474

**Published:** 2022-06-16

**Authors:** MaKayla F. Cox, Erin R. Hascup, Andrzej Bartke, Kevin N. Hascup

**Affiliations:** ^1^ Dale and Deborah Smith Center for Alzheimer’s Research and Treatment, Department of Neurology, Neurosciences Institute, Southern Illinois University School of Medicine, Springfield, IL, United States; ^2^ Department of Pharmacology, Southern Illinois University School of Medicine, Springfield, IL, United States; ^3^ Department of Internal Medicine, Southern Illinois University School of Medicine, Springfield, IL, United States; ^4^ Department of Medical Microbiology, Immunology and Cell Biology, Southern Illinois University School of Medicine, Springfield, IL, United States

**Keywords:** excitotoxcity, neurodegenerative disease, amyloid—beta, geroscience, biomarker, hippocampus, neuroimaging, growth hormone receptor knockout

## Abstract

Aging is a naturally occurring decline of physiological processes and biological pathways that affects both the structural and functional integrity of the body and brain. These physiological changes reduce motor skills, executive function, memory recall, and processing speeds. Aging is also a major risk factor for multiple neurodegenerative disorders including Alzheimer’s disease (AD). Identifying a biomarker, or biomarkers, that signals the transition from physiological to pathological aging would aid in earlier therapeutic options or interventional strategies. Considering the importance of glutamate signaling in synaptic plasticity, motor movement, and cognition, this neurotransmitter serves as a juncture between cognitive health and disease. This article discusses glutamatergic signaling during physiological aging and the pathological changes observed in AD patients. Findings from studies in mouse models of successful aging and AD are reviewed and provide a biological context for this transition. Finally, current techniques to monitor brain glutamate are highlighted. These techniques may aid in elucidating time-point specific therapeutic windows to modify disease outcome.

## Graphical Abstract

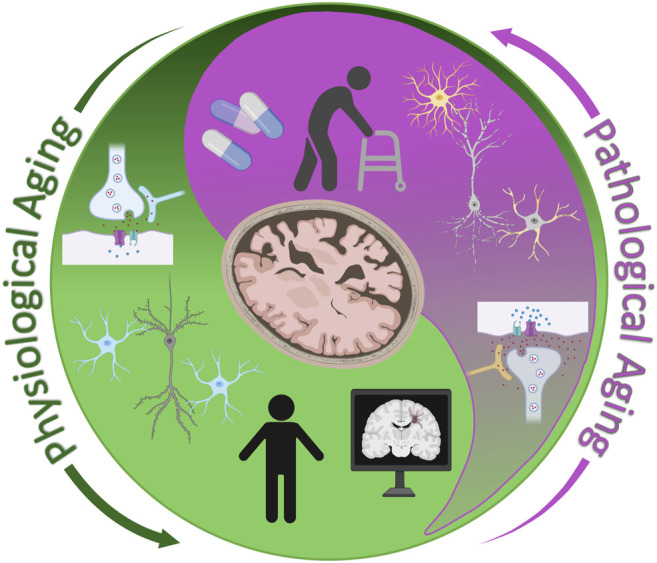

## Introduction

Aging is a complex biological process accompanied by declines in physiological function leading to increased susceptibility for disease and death (Carmona and Michan 2016). Aging is inevitable, occurring in all organisms with time. Over the past decades, innovative improvements in medicine have extended human life span and prolonged the aging process. But, a concomitant improvement to geriatric health has not followed. This unprecedented aging of the human population in developed nations has resulted in people living with multiple chronic health conditions (Barzilai et al., 2018). Maintaining their quality of life has created a significant socio-economic burden on both family members and governments. Accordingly, the geroscience hypothesis postulates that slowing the rate of biological aging can prevent, delay, or ameliorate the development of these chronic conditions leading to successful aging.

Research over the past decades has elucidated nutritional, genetic, and pharmacological interventions that can increase life span of numerous model organisms such as yeast, *Caenorhabditis elegans*, *Drosophila melanogaster*, and mammals. Some of these life span extension mechanisms have shown therapeutic potential in numerous model organisms of neurodegenerative disorders including Alzheimer’s disease (AD) (Soo et al., 2020). These beneficial effects have translated poorly into human clinical trials. By the time chronic illness becomes evident, the biological mechanisms governing healthy aging have succumbed to the chronic condition. Intervening years before the onset of chronic illness is ideal, but involves individual life style modifications such as diet and exercise. Despite their known health benefits long term adherence to these regimens is poor. Identifying the juncture when physiological aging transitions into a pathological disease state provides an opportune pharmacological window to modify disease outcome before onset of clinical symptoms.

Biomarkers are becoming increasingly common for use in understanding the physiological processes that occur with age and disease onset, (Crimmins et al., 2008). Biomarkers are molecules present in blood, body fluid, and tissues that can be quantitatively measured to directly assess disease onset or progression. The continuous progression of molecular diagnostics allows for accurate detection and quantification of biomarkers for various uses. Biomarkers have been separated into two types: biomarkers of exposure and biomarkers of disease. Our laboratory focuses on biomarkers of disease with a goal of earlier diagnosis and tighter monitoring of disease progression (Mayeux 2004). Within biomarkers of disease are functional biomarkers which provide a direct measurement of target engagement. In order to use a biomarker, there must be a distinguishable quantifiable change in the amount of the marker present in the healthy versus diseased population. For example, amyloid-β_42_, phosphorylated tau, and neurofilament light chain provide diagnostic utility when used in concert to evaluate AD progression (Jack et al., 2016). Although these proteins provide an indication of disease severity, they do not indicate the initial change from physiological aging to pathological disease. We propose examination of central nervous system (CNS) glutamate levels as a functional biomarker due to its role in procognitive changes before mild cognitive impairment (MCI) and eventual onset of AD. The focus of this article is to discuss changes in glutamatergic regulation as it relates to aging and AD. Based on our work and the research of others, we believe monitoring glutamate dynamics as a viable biomarker for identifying the conversion from healthy physiological aging to pathological dysfunctional aging.

## Overview of Glutamatergic Neurotransmission

Glutamate is the most abundant neurotransmitter in the CNS with glutamate receptors present on more than 90% of neurons and 40% of synapses (Bukke et al., 2020; Conway, 2020; Gasiorowska et al., 2021). Currently, there are over 20 glutamate receptors identified within the CNS with each individual receptor having multiple subtypes (Pankevich et al., 2011). Glutamate homeostasis is critical for healthy aging and reducing the risk for various neurological diseases including epilepsy, addiction, amyotrophic lateral sclerosis, Parkinson’s disease, AD, and more (Scofield et al., 2016; Alcoreza et al., 2021). Physiologically, glutamate plays a critical role in synaptic stability and plasticity (Tapiero et al., 2002). The functional elements encompassing glutamate neurotransmission are the pre and postsynaptic neurons along with glial cells which are conjunctionally defined as the “tripartite synapse,” (Halassa et al., 2007; Lalo et al., 2021). The tripartite synapse functions through both metabotropic and ionotropic receptors. A comprehensive review of these receptors and their functions are detailed elsewhere (Findley et al., 2019) and briefly discussed below.

Glutamate is synthesized from glutamine by glutaminase in presynaptic neurons where it is then transported to synaptic terminals (Revett et al., 2013). While in the synaptic terminal, glutamate is stored into vesicles by vesicular glutamate transporters (VGLUT) 1–3 for immediate release upon neuronal depolarization (Takamori et al., 2000; Fremeau et al., 2004). Signaling is mediated through ionotropic and metabotropic glutamate receptors (iGluR and mGluR) located throughout the tripartite synapse. Signal transduction is terminated by uptake into high-efficiency excitatory amino acid transporters (EAATs) which are predominantly located on astrocytes. Once in the astrocyte, glutamine synthetase converts glutamate into glutamine where it can be released back into the extracellular space for return to the presynaptic terminal in a process referred to as the glutamate/glutamine cycle. Together, iGluR, mGluR, and EAATs act as critical regulators of the glutamatergic system through modulating signal strength and extracellular concentrations of glutamate. Meticulous regulation of extracellular glutamate is crucial to prevent persistent receptor activation that can result in excitotoxicity and neuronal loss.

iGluRs are formed by heterotetrametric and homotetrameric subunits and are voltage sensitive and fast acting due to the influx of ions across the plasma membrane (Pankevich et al., 2011). These receptors are expressed both pre- and post-synaptically (Wisden and Seeburg 1993). There are three subtypes of ionotropic receptors: N-methyl D-aspartate (NMDAr), α-amino-3-hydroxy-5-methyl-4-isoxazolepropionic acid (AMPAr), and kainate receptors. The NMDAr and AMPAr are colocalized on the postsynaptic membrane as they are functionally dependent on one another during membrane depolarization (Takumi et al., 1999). The quick activation of AMPAr facilitates the NMDAr by depolarizing the membrane and allowing for NMDAr to overcome the Mg^2+^ blockade. However, NMDAr and AMPAr interact with glutamate independently. NMDAr are comprised of subunits that provide different functions. The NR2A and NR2B subunits bind with glutamate and have been shown to mediate excitotoxicity in cultured cortical neurons (Engelhardt et al., 2007). The high density of NMDAr expressed on neurons and astrocytes throughout the brain, and most notably in the hippocampus, contribute to learning, memory, and synaptic plasticity (Barco et al., 2006; Lee et al., 2010), but overactivation drives neuronal loss. This dichotomy is due to the cellular localization of these receptors. Synaptic NMDAr activation drives calcium-mediated transcriptional changes to promote neuronal health and resistance to cellular insults. Extrasynaptic NMDAr activation is coupled to multiple signaling pathways that reduces stress resistance and drives apoptosis which may be an initiating factor for multiple neurodegenerative disorders (Hardingham and Bading, 2010).

AMPAr interact with glutamate through four subunits (GluA_1_–GluA_4_) that are highly homologous with conserved transmembrane and extracellular domains (Collingridge et al., 2004; Chater and Goda 2014). Presynaptically, AMPAr function to promote synapse and spine formation (Isaac et al., 2007). The functions of AMPAr in learning and memory are not well characterized, however, AMPAr are suspected to play a role in synaptic plasticity through augmentation of Ca^2+^ entry into NMDAr (Chater and Goda 2014). AMPAr are also important for potentiating other receptors in the cell signaling process. iGluRs are important for mediating rapid neuronal communication whereas metabotropic receptors gradually mediate changes through signal transduction pathways.

mGluRs are slow acting signal transducers present on both presynaptic and postsynaptic neurons (Revett et al., 2013). There are three subgroups of mGluRs distinguishable by their pharmacological and signaling properties. Group I is comprised of postsynaptically expressed Gq-coupled receptors while Groups II and III are Gi/o-coupled inhibitory auto receptors expressed both presynaptically and postsynaptically (Petralia and Wenthold 1996; Rudy et al., 2015). Dysregulated mGluR signaling contributes to anxiety, depression, learning impairments, cognitive decline, and choreas. mGluR1 and mGluR5 are identified as Group I receptors and facilitate the activity of various kinases (Ménard and Quirion 2012). Activation of mGluR1 and mGluR5 has also been shown to induce long term depression (LTD), a type of synaptic plasticity that decreases responsiveness to glutamate. Therefore, Group I receptors are important for memory formation and learning processes (Kang and Kaang 2016). Group II receptors include mGluR2 and mGluR3. These receptors are inhibitory autoreceptors and play a critical role in glutamate regulation as they are situated both presynaptically and postsynaptically (Hascup et al., 2012). Group III receptors are comprised of mGluR4, mGluR7, and mGluR8 in the cerebrum. These three receptors have been established as neuroprotectants with the capability to induce both long-lasting potentiation of glutamatergic transmission and inhibition of glutamatergic transmission (Neugebauer, et al., 1997; Schrader and Tasker 1997; Grueter and Winder 2005). The dual location of mGluRs facilitates both suppression and increased glutamate signaling (Swanson et al., 2005; Lesage and Steckler 2010). The extensive distribution of mGluRs can help to monitor extracellular glutamate levels and modulate the glutamatergic tone. Therefore, the conservation of iGluRs and mGluRs and their ability to modulate the glutamatergic system across aging are important for maintaining cognitive health.

## Physiological Aging

Aging is a naturally occurring decline of physiological processes modifying various biochemical pathways throughout the life span of all organisms (Bartke 2021). Physiological declines in motor movement, metabolism, inflammation, and executive function are prevalent and vary between individuals and sexes (Peters 2006). These declines are attributed to numerous factors including sarcopenia, adiposity, and arteriosclerosis. Aging also affects both structural and functional integrity of the brain contributing to these physiological declines.

## Glutamate Regulation in Physiological Aging

The abundance of glutamate and glutamate receptors present in the brain contribute to the desire to understand how the glutamatergic system can contribute to the aging processes. Brain morphological changes such as cortical thinning and synaptic pruning are important aspects of brain development, but these become more pronounced with age leading to altered glutamatergic neurotransmission (Segovia et al., 2001; Anderton 2002; Brans et al., 2010). These age-related changes in glutamate occur in the hippocampus, prefrontal cortex, and motor and sensory areas (Segovia et al., 2001; Kaiser et al., 2005; Raininko and Mattsson 2010) that helps to explain the subsequent cognitive, motor, and sensory decline in healthy aging.

During healthy aging, there is general agreement that the total pool for neuronal glutamate signaling decreases (Gasiorowska et al., 2021). Examination of healthy individuals between the ages of 24 and 68 found that older subjects had lower glutamate concentrations in the motor cortex as compared to younger subjects (Kaiser et al., 2005). Similar age-related glutamate decline was seen in the striatum but not the pons or cerebellum (Zahr et al., 2013). Two meta-analyses of healthy aging humans using magnetic resonance spectroscopy (MRS) revealed that brain glutamate concentrations decrease in conjunction with brain volume shrinkage (Roalf et al., 2020; Sydnor and Roalf 2020). These physiological changes may start by midlife. Glutamate concentrations decreased 0.33 mM/year in the medial frontal cortex of 18–31 years-old study subjects (Marsman et al., 2013). This decline occurred in combination with grey matter thinning, but these anatomical changes may not solely explain decreased glutamate levels. Additionally, men more than women may be more affected by age-related brain glutamate decreases (Sailasuta et al., 2008).

Despite the importance of glutamate signaling in health and disease, this has yet to be evaluated in the oldest-old. Nonagenarians and centenarians have demonstrated a resiliency against the onset of age-related disease (Engberg et al., 2009) and have been proposed as the optimal study participants to identify genes related to successful aging (Marcos-Pérez et al., 2021). A majority of these individuals have normal cognitive and functional ability (Sachdev et al., 2013). This suggests glutamate levels decrease at a slower rate or other compensatory mechanisms occur to modulate signaling.

## Glutamate Regulation in Animal Models of Normal Aging

Similar to what has been observed in humans, rats and mice show a subtle decrease in glutamatergic synapses and neurons during aging—particularly when compared to pathological conditions (Temido-Ferreira et al., 2019; Gasiorowska et al., 2021). This causes an age-related shift in synaptic plasticity that favors LTD over LTP (Kumar and Foster, 2019) that contributes to the natural cognitive decline consistently observed in multiple species across numerous behavioral tasks (Tanila et al., 1997). Over the past 40 years researchers have studied hippocampal glutamatergic changes to disentangle aging effects from disease progression. Unless specifically stated, the studies discussed below use rodents that were at least 24 months old. Hippocampal glutamate protein content is reduced in Fisher 344 rats (Banay-Schwartz et al., 1989) and in the temporal cortex of Sprague-Dawley rats (Jing et al., 2016). This lower protein content contributes to reduced tonic and stimulus-evoked glutamate release observed in the Fisher 344 rat model of aging (Zhang et al., 1991; Stephens et al., 2011). Similar findings are routinely reported in aging mouse models. C57BL/6 mice have reduced neurometabolic activity and hippocampal glutamatergic neurotransmitter cycling (Bahadur Patel et al., 2021) along with reduced VGLUT1 protein concentration (Rozycka et al., 2019). This longitudinally decreases cortical, striatal, and hippocampal glutamate in both sexes of C57BL/6 mice as measured by MRS (Duarte et al., 2014). Evoked hippocampal glutamate release is reduced in 17 months old C57BL/6J mice compared to younger (7 months) littermates (Minkeviciene et al., 2008). Changes to glutamatergic receptor density are also prominent in aged rats, mice, and nonhuman primates. 25–32 year old rhesus macques show subregion specific declines in NMDAr subtype expression that correlates with cognitive deficits (Hara et al., 2012). Hippocampal NMDAr and AMPAr density was decreased in Fisher 344 rats while EAATs remained consistent (Mitchell and Anderson, 1998; Segovia et al., 2001). Additionally, glutamate binding to NMDAr decreases with age in C57BL/6 and BALB/c mice (Peterson and Cotman, 1989). The decreased glutamatergic signaling across species can be interpreted as a biologically conserved mechanism of aging similar to sarcopenia or osteoporosis. Accordingly, this decline should be blunted or delayed in models of successful aging.

## Glutamate Regulation in Animal Models of Longevity

Our laboratory is interested in the role of glutamatergic signaling in healthy aging and how changes can drive the transition to neurodegenerative disorders. Animal models of successful aging are important in biogerontology research for understanding mechanistic factors that contribute to the aging process. Suppression of the growth hormone/insulin-like growth factor 1/insulin axis is known to positively influence life span and health span through pleiotropic factors including protection against neurodegeneration. Mice with disruption of the growth hormone receptor (GHRKO) have reduced insulin-like growth factor 1 and elevated growth hormone that results in a diminutive phenotype with exceptional longevity. Disruption of this signaling pathway also increases neuronal differentiation and increases cortical neuronal density (Turnley et al., 2002; Ransome et al., 2004). Examination of CNS tissue by our laboratory revealed conserved or reduced expression of glutamatergic markers as GHRKO mice age (Hascup et al., 2015). In particular, hippocampal VGLUT1, GluN2B, and GluA1 expression levels decline with age that may provide GHRKO mice protection against excitotoxicity. In a follow up study, we examined hippocampal glutamatergic dynamics in 20–24 months old GHRKO and littermate control mice. To monitor *in vivo* glutamate our laboratory uses an enzyme-based microelectrode array with high spatial (micron) and temporal (millisecond) resolution that cause minimal damage to the surrounding parenchyma (Hascup et al., 2009; Hascup et al., 2010). We found that aged GHRKO mice maintained cognition and glutamate signaling throughout the hippocampus compared with age-matched littermate controls (Hascup et al., 2016). The GHRKO memory retention at advanced age was similar to previous studies (Kinney et al., 2001a) and may suggest the delayed cognitive aging in these mice is conferred through glutamatergic regulation.

Ames Dwarf mice are homozygous deficient in the Prophet of Pituitary Factor 1 gene an upstream regulator of Pituitary Factor 1 that confers deficiency in growth hormone, prolactin, and thyroid stimulating hormone. Similar to GHRKO mice, this mutation results in a diminutive size with an 1 year average lifespan extension compared to littermates (Brown-Borg et al., 1996). At 3 months of age, Ames Dwarf mice have higher hippocampal NMDAr expression (Sharma et al., 2012) and cAMP response element-binding protein (Sun et al., 2005) that may contribute to their enhanced learning and memory at older ages (Kinney et al., 2001b). Together, these animal models of successful aging may suggest that healthy cognitive aging requires maintenance of glutamatergic signaling throughout the aging process. When disruption occurs, the mechanisms of healthy aging are no longer conserved and may precipitate the onset of neurodegenerative disorders (Li et al., 2021).

## Pathological Aging

Deviations from healthy aging to pathological aging are the driving force of age-related disease. The risk for cardiovascular diseases, cancer, neurodegenerative diseases, autoimmune diseases, and musculoskeletal issues increases significantly with age (Li et al., 2021). Therefore, understanding the mechanisms behind physiological aging could facilitate the direction of future studies on the biological drivers of pathological aging. For example, EAAT density and function decrease with age (Segovia et al., 2001) leading to extrasynaptic glutamate spillover. Activation of extrasynaptic glial NMDAr is a contributing factor to excitotoxicity during pathological aging. Our laboratory focuses on the glutamatergic system and its contributions to AD. We propose alterations in brain glutamate concentrations as a critical proponent for the pathological aging processes.

## Alzheimer’s Disease

AD is an age-related neurodegenerative disorder characterized by progressive anterograde amnesia and the seventh leading cause of death in the US (Ahmad and Anderson 2021). Aging is the primary risk factor for developing AD, with 65 years of age designated as the cutoff that defines early-onset versus late-onset AD. Only 2%–10% of cases are categorized as early-onset (Cacace et al., 2016) with one in nine individuals aged 65 years or older diagnosed with AD (Rajan et al., 2021). Currently, an estimated 5.8 million Americans are diagnosed with AD and this number is projected to double by 2050 (Alzheimer’s Association, 2020) posing a significant socioeconomic burden. Despite onset differences, the structural pathologies are similar with hippocampal atrophy being the most prominent shared feature (Eckerström et al., 2018). The mechanisms behind development of cognitive deficits are numerous, but research has predominantly focused on accumulation of misfolded protein aggregates.

The deposition of amyloid beta (Aβ)_42_ that aggregates into extracellular plaques and hyperphosphorylated tau protein that misfolds into intracellular neurofibrillary tangles (NFT) are the hallmark pathological changes typically observed in postmortem tissue. These proteinopathies are hypothesized to begin abnormally accumulating in the brain 10–20 years prior to the onset of overt symptoms (Giannakopoulos et al., 1995; Bateman et al., 2012; Beason-Held et al., 2013). Various genetic and environmental components have been identified as risk factors contributing to these proteinopathies (Hascup and Hascup 2020). The complexity of AD has made early detection and treatment difficult. Current treatments are symptomatic with discordant results towards disease modification for next generation medications focused on plaque removal. This lack of efficacy is often attributed to poor biomarker identification for earlier interventional strategies. Current biomarkers do not adequately identify asymptomatic AD nor disease progression making it difficult to provide disease-stage specific treatments for optimal efficacy. The identification of proteins associated with changes occurring prior to the onset of symptoms could lead to the potential to develop timepoint dependent therapeutics for improved specificity.

## Glutamate Dysregulation in Alzheimer’s Disease Patients

Various imaging techniques and postmortem tissue analyses have shown dysregulation of the glutamatergic system throughout the tripartite synapse. These changes vary across the continuum of cognitive impairment in AD and are largely affected by protein accumulation and aggregation status. Initially, either Aβ or hyperphosphorylated tau contributes to neuronal excitability that facilitates overexcitation and in turn leads to glutamatergic excitotoxicity (Huijbers et al., 2019; 2015). Hyperactivity along with morphological changes in neuronal structure are thought to be early contributors to AD pathology and cognitive dysfunction (Ghatak et al., 2019). The persistent overactivation of NMDAr perturbs recognition of physiological signals thereby affecting memory consolidation and recall (Danysz and Parsons, 2012). Later stages of AD are contraindicative of hyperactivity and demonstrate markedly decreased glutamate levels. This is attributed to neuronal loss caused by either hyperphosphorylated tau microtubule destabilization or the exposure to an excitotoxic environment.

Multiple receptors and transporters are responsible for altered glutamate signaling that show biphasic changes across the AD continuum. Presynaptically, soluble Aβ preferentially binds to VGLUT1 potentiating release characteristics in the early stages (Sokolow et al., 2012). While decreased VGLUT1 levels have been found in later stages of post-mortem brain tissue (Kirvell et al., 2006; Rodriguez-Perdigon et al., 2016). mGluR2 expression was increased in hippocampal pyramidal neurons and associated with hyperphosphorylated tau deposition in postmortem AD tissue (Lee et al., 2004). Considering the autoinhibitory nature of Group II receptors on synaptic signaling, this increase may be a compensatory mechanism to attenuate extracellular glutamate levels. Postsynaptically, AMPAr and NMDAr subunits change during the AD continuum with upregulation seen early in disease progression followed by downregulation in later disease stages (Findley et al., 2019). These changes may serve to modulate the strength of synaptic signaling to maintain cognitive processing during receptor over activation. mGLUR5 is ubiquitously expressed throughout the medial prefrontal cortex that potentiates NMDAr function and expression. Aβ_42_ complexes to mGluR5 along with cellular prion protein (Rammes et al., 2011; Piers et al., 2012). This binding disrupts multiple signal transduction pathways (Abd-Elrahman and Ferguson, 2022) that subsequently reduces receptor expression in later disease stages (Mecca et al., 2020). Glial EAATs are both downregulated (Jacob et al., 2007; Hoshi et al., 2018; Kobayashi et al., 2018) and have reduced transporter capacity due to altered splice variants (Scott et al., 2011). Finally reduced γ-aminobutyric acid neuronal density tips the excitatory/inhibitory scale to favor glutamate release (Palop et al., 2007; Lauterborn et al., 2021).

### Glutamate Dysregulation in Alzheimer’s Disease Animal Models

Accumulation of Aβ_42_ is hypothesized to begin decades prior to cognitive decline (Sperling et al., 2014) These soluble isoforms are considered the neurotoxic species associated with AD progression and have been shown to bind to the alpha 7 nicotinic acetylcholine receptor (α7nAChR) (Wang et al., 2002). Our laboratory and others have shown that binding of soluble Aβ_42_ to this receptor elicits glutamate release (Mura et al., 2012) from presynaptic receptors (Hascup and Hascup 2016). Within the hippocampus, the dentate (DG) and CA1 had the largest responses to the lowest concentrations of Aβ_42_ applied. Considering our laboratory is focused on understanding glutamatergic dynamics during aging and its dysregulation in disease, we have strived to understand these changes throughout disease progression using an animal model of AD.

The APP/PS1 mouse is a transgenic amyloidogenic AD model that initially develop plaque pathology and subtle cognitive deficits at 6 months. By 12 months of age these mice have plaques prevalent throughout the hippocampus and observable cognitive deficits (Webster et al., 2014). While fast progressing AD models reduce study duration, the slower progression of both pathology and cognitive decline in APP/PS1 mice and other models is ideal for several reasons. First, the slower onset of amyloid deposition allows mice to naturally age. This is more conducive to identifying dysregulated aging processes that contribute to disease progression. Second, we can design early interventional strategies without potential confounds from developmental biology that have a greater potential to occur with faster progressing models. Third, we can identify windows of disease progression for improving the translational relevance of disease-stage specific interventional strategies. Based on the pathology and cognitive decline in the APP/PS1 mice compared to their littermate control C57BL/6 mice, 6 months of age corresponds with mild cognitie impairment while 12–15 and 18+ months correspond with mild-AD and AD, respectively.

We have shown that hippocampal basal and stimulus-evoked glutamate signaling in APP/PS1 mice becomes hyperactive in a subregion specific manner prior to onset of cognitive decline (Hascup and Hascup 2015). The first changes are seen in the CA1 where stimulus-evoked glutamate release is markedly elevated by 3 months of age. At 6 months, when hippocampal plaque accumulation begins, basal glutamate is elevated throughout the hippocampus and continues to increase with age. Glutamate release also increases in the DG at this age and stays elevated throughout disease progression. CA3 evoked glutamate release does not become elevated until 12 months of age when plaque accumulation is more prominent (Hascup et al., 2020a). We also observed that plaque deposition was anatomically aligned, but temporally delayed with hyperactive glutamate signaling (Hascup et al., 2020b), further supporting a role of the glutamatergic system as a potential early biomarker and therapeutic target. An increase in Aβ_42_ with age provides mechanistic insight into the temporal profile of hippocampal hyperglutamatergic signaling. In summary, dysregulation of glutamatergic signaling followed a subregion specific pattern with the CA1 > DG > CA3; not unlike the sensitivity of these regions to locally applied soluble Aβ_42_ (Hascup and Hascup 2016).

**FIGURE 1:**
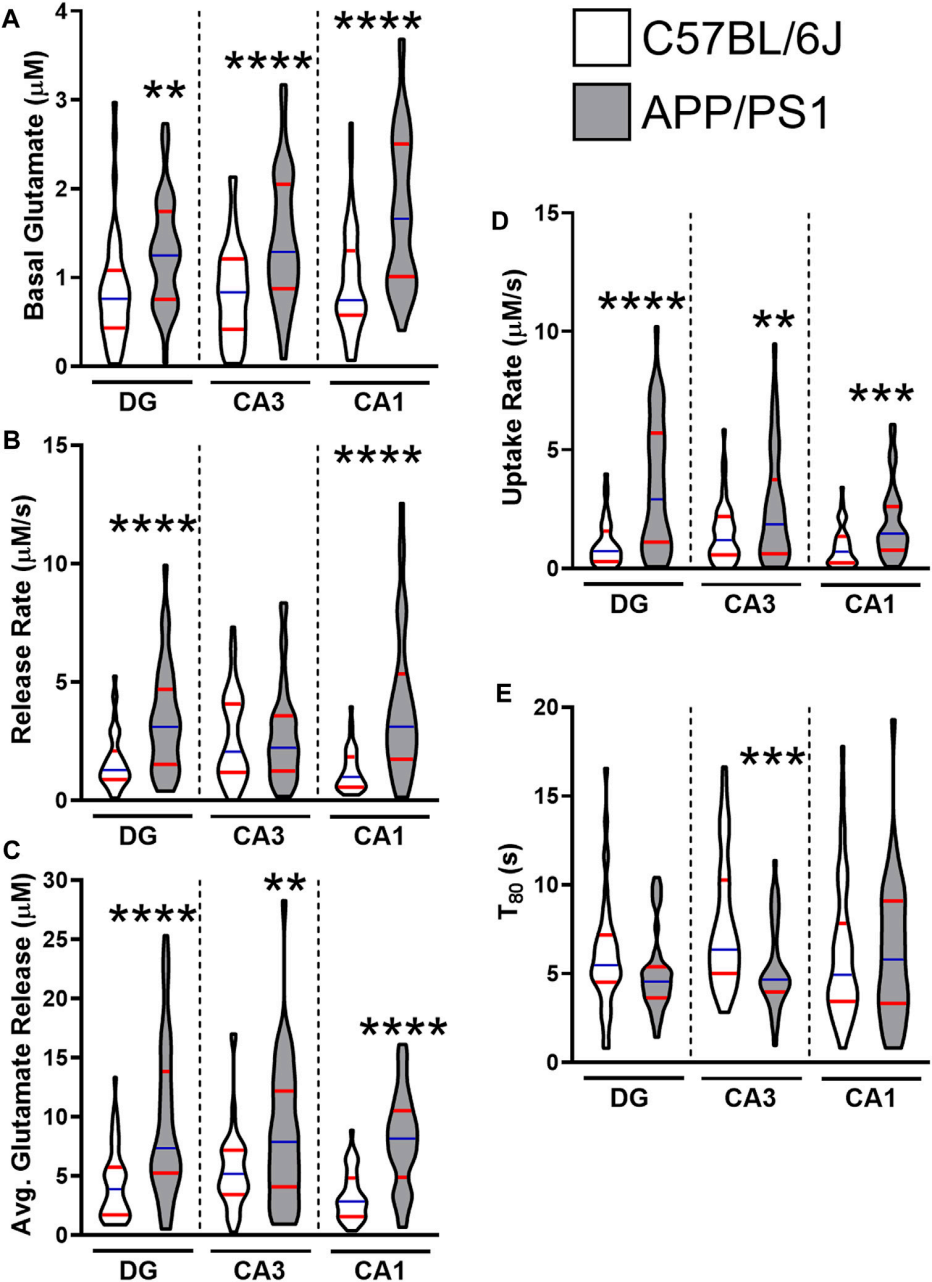
Glutamate dynamics in APP/PS1 and C57BL/6J mice at 12 months. The figures were created using control 12–15 months male C57BL/6J (white) and APP/PS1 (gray) mice across multiple datasets (Hascup E. R. et al., 2019; Hascup K. N. et al., 2019; Hascup et al., 2020a). Graphs depict violin plots with median (blue line) and quartiles (red line). **(A)** Basal glutamate levels were measured in the CA1, CA3, and the DG of the hippocampus using a microelectrode array (MEA). **(B)** Glutamate release rate was calculated using the change in amplitude between the maximal response and baseline over the duration (s) to reach maximal response after stimulation. **(C)** Average glutamate release was determined using the maximal change after stimulation from baseline. **(D)** Glutamate clearance followed first-order-rate kinetics. A logarithmic slope for glutamate concentration decay (k_−1_) versus time (s^−1^) is estimated using regression analysis (R^2^ ≥ 0.9) to determine the uptake rate. **(E)** T_80_ refers to the duration of time needed for 80% of the maximal glutamate signal to be cleared from the extracellular space. A two tailed *t*-test was used to compare genotypes in each hippocampal subregion. **p < 0.01, ***p < 0.001, ****p < 0.0001; *n* = 36–47.

By 12 months, all regions of the APP/PS1 hippocampus routinely show hyperglutamatergic signaling. Figure 1 provides a grouped comparison of control APP/PS1 and age-matched C57BL/6J cohorts taken with multiple studies from our laboratory. The graphs depict the reproducibility of elevated hippocampal basal glutamate and stimulus-evoked release (Figures 1A–C) across multiple published datasets. We have also observed that APP/PS1 mice have a compensatory increase in glutamate clearance (Figures 1D,E) suggesting either an upregulation to transporter density or function. Despite the increased clearance, this still fails to modulate glutamate to nonpathological levels. Besides the aging associated changes to Aβ_42_, we have also shown VGLUT1 density is elevated at 12 months in APP/PS1 mice (Hascup E. R. et al., 2019). When taken together, the increases to both presynaptic stimulation and glutamatergic vesicles accounts for the elevated levels observed.

A couple of limitations should be considered when interpreting these results. First, these studies were conducted only in male mice. Others have shown that stimulus-evoked hippocampal glutamate was decreased at 17 months of age in female APP/PS1 mice (Minkeviciene et al., 2008). While this was similar to our observations in the CA1 of male APP/PS1 mice, additional research is required to determine if the glutamatergic signaling profile differs during disease progression between sexes of APP/PS1 mice. Second, we have only assayed glutamate dynamics in a single model of cerebral amyloidosis. Multiple laboratories have reported on these changes in other amyloid and tau AD models, which are discussed below. To further address these limitations, our laboratory is actively probing sex as a biological variable to understand hippocampal glutamate changes throughout disease progression of additional AD models such as the APP^NL-F/NL-F^ mice.

Similar glutamate signaling changes during disease progression have been reported in other AD animal models and summarized in Table 1. The rTg4510 mice express human tau containing the P301L mutation. This model develops neurofibrillary tangle, neuronal loss, and age-dependent cognitive deficits. By 6 months, these mice also have elevated hippocampal glutamate that correlated with acquisition errors on the Barnes maze behavioral task (Hunsberger et al., 2014; Hunsberger et al., 2015). Hippocampal glutamate levels are also higher at 3 months of age in the PS19 tauopathy AD model. As these mice continue to age, the number of synapses, and neurons decrease causing a corresponding reduction in glutamate levels. The elevated glutamate early in life coupled with neuronal loss at later stages suggests an excitotoxic mechanism (Crescenzi et al., 2017). However, it should be noted that the human P301L transgene insertion disrupts an endogenous mouse gene that contributes to the molecular changes and cognitive decline observed in this model (Gamache et al., 2019). The 3xTg AD model contains familial mutations to the amyloid precursor protein, presenilin 1, and microtubule associated protein tau genes resulting in dual amyloid and tau pathology by 12 months of age. Persistent hyperactive glutamatergic synapses were detected using electrophysiological techniques in the entorhinal cortex at this age (Arsenault et al., 2011). 5xFAD mice have multiple transgene insertions for human amyloid precursor protein and presenilin 1 that causes rapidly progressing amyloid accumulation starting at 6 weeks of age. Whole-cell patch clamp recordings of CA1 pyramidal neurons show higher spontaneous excitatory post synaptic currents at 2 and 4 months of age (Li et al., 2021). All together these studies show that hyperactive glutamatergic signaling either precedes or coincides with amyloid and/or tau accumulation.

**TABLE 1:** The onset of pathological, cognitive and glutamatergic changes in AD mouse models.

Model	Mutations	Pathology onset	Cognitive decline onset	Hyperactive glutamate signaling onset	Reference
APP/PS1	APP KM670/671NL	Plaques: 4–6 mos	6–10 mos	CA1: 2–3 mos	Hascup et al. (2020b)
	PSEN1dE9			DG & CA3: 6–8 mos	
5xFAD	APP KM670/671NL	Plaques: 2 mos	3–6 mos	CA1: 2.5 mos	Li Y. et al. (2021)
	APP I716V				
	APP V717I				
	PSEN1 M146L				
	PSEN1 L286V				
3xTg	APP KM670/671NL	Plaques: 6 mos	4 mos	Entorhinal Cortex: 12 mos	Arsenault et al. (2011)
	PSEN1 M146V				
	MAPT P301L	Tangles: 12 mos			
rTg4510	MAPT P301L	Tangles: 2.5–4 mos	2.5–4 mos	DG, CA1, and CA3: 5–7 mos	Hunsberger et al., 2014, 2015
Tau P301S (Line PS19)	MAPT P301S	Tangles: 6 mos	6 mos	Hippocampus: 3 mos	Crescenzi et al. (2017)

The build-up of pathological proteins during disease progression may initiate the transition from low glutamate levels that are observed in healthy aging. These temporal hippocampal glutamate changes could serve as a biomarker for identifying a switch between physiological and pathological aging. Understanding this progression would help provide disease-stage specific interventions. For example, we treated APP/PS1 mice with riluzole, a glutamate modulator, starting when we first observed changes in the CA1. This treatment not only attenuated both basal and evoked glutamate release, but also provided procognitive effects (Hascup et al., 2020a). Other laboratories have shown these effects are modulated through EAAT2 as well as NMDAr subunit expression levels (Pereira et al., 2017; Okamoto et al., 2018).

## Diagnostic Methods for *In Vivo* Glutamate Monitoring

Increasing evidence supports the importance of modulating glutamatergic signaling as a therapeutic treatment for AD. Therefore, methods to monitor glutamate as it transitions from physiological to pathological signaling are needed. The following sections discusses minimally invasive and non-invasive techniques that can be utilized in both humans and preclinical AD models.

Cerebrospinal fluid (CSF) provides an indication of the brain parenchyma and is currently used to measure deposition of amyloid and tau while determining neuronal loss by neurofilament light chain. Samples are collected and analyzed after a lumbar puncture commonly referred to as a spinal tap. During this 30–40 min procedure, the patient is given a local anesthetic before a needle is inserted between two lumbar vertebrae to collect CSF. The lumbar puncture can be difficult to perform and leave post-procedural issues including a “spinal headache.” Several studies have analyzed glutamate in the CSF of MCI and AD patients indicating increases or decreases compared to healthy aging (Madeira et al., 2018). Rather these discordant results may reflect progression through the AD continuum. While lumbar punctures are minimally invasive, they have the benefit of being 3–4 times less expensive than the imaging techniques discussed below (Tariciotti et al., 2018).

Currently, advanced neuroimaging methods are being explored to detect changes in glutamate receptors and glutamate excitation in healthy aging, MCI, and AD patients. Imaging offers the potential to detect changes in metabolic functions within the brain and monitor structural transformations throughout the aging process. Positron emission tomography (PET) imaging can be used with multiple radiotracers to quantitatively assess synaptic density. Radiotracers are molecules where one or more atoms are replaced with a radioactive isotope and their *in vivo* decay is monitored. Radiotracers can be created to target specific receptors in the brain that can be visualized using gamma ray emissions (Schaffer et al., 2015). Currently, PET imaging is used to assess multiple AD pathophysiological targets including amyloid, tau, neuronal density, and neurotransmitter signaling—including glutamate (Bao et al., 2021). Using radiotracers targeting mGluR5 researchers found decreased hippocampal expression in early AD patients that correlated with lower episodic memory scores and decreased global function (Mecca et al., 2020; Treyer et al., 2020). The small number of glutamate signaling radiotracers, short decay times, and large financial costs all limit the clinical utility of PET imaging.

Magnetic resonance (MR) uses a magnetic field to create a three-dimensional image of the brain without the need for ionizing radiation or radiotracers. Unlike PET imaging, MR is unable to directly measure neural activity (Osborne et al., 2015). MRS is capable of detecting the chemical composition of the tissue by examining the magnetic moment of nuclei such as protons (^1^H) and carbons (^13^C). MRS has allowed for the noninvasive quantification of regional neurotransmitters including glutamate in numerous diseases and preclinical disease models. Multiple MRS studies have shown decreased hippocampal glutamate concentrations in AD patients (Lin et al., 2003; Fayed et al., 2011; Graff-Radford and Kantarci, 2013).

In the last decade MR has been used in conjunction with chemical exchange saturation transfer (CEST) allowing for the indirect detection of brain metabolite changes using endogenous proteins. CEST uses exchangeable amide protons to monitor glycogen, glycosaminoglycans, γ-aminobutyric acid, and glutamate (GluCEST) (Kogan et al., 2013). GluCEST has been validated in humans (Nanga et al., 2018) and is capable of sub-hippocampal measurements. The majority of GluCEST studies, however, have focused on preclinical aging and disease models. Aging reduced GluCEST contrast in multiple networks associated with cognition in mouse lemur primates (Garin et al., 2022). Decreased hippocampal glutamate has also been observed in 18+ month old amyloid (Haris et al., 2013) and tau (Crescenzi et al., 2014) mouse models of AD similar to observations using implantable biosensors. Considering GluCEST is noninvasive, longitudinal analysis in mouse models would provide initial clinical utility for examination across the AD continuum in humans.

## Conclusion

The extensive amount of knowledge accumulated through decades of research on the biology of glutamate leaves this system poised to make major advancements in the realm of neurological disorders. Our laboratory has shown the potential for using glutamate as a biomarker for determining the juxtaposition between physiological and pathological aging. Advances using minimally and noninvasive techniques allows for longitudinally monitoring regional changes in glutamate levels. These methods have the potential to provide disease state dependent therapeutic interventions for improving patient outcome.

## Abbreviations

α7nAChR, α7 nicotinic acetylcholine receptor; AMPAr, α-amino-3-hydroxy-5-methyl-4-isoxazolepropionic acid receptors; AD, Alzheimer’s disease; Aβ, amyloid-β; CR, caloric restriction; CNS, central nervous system; DG, dentate gyrus; EAAT, excitatory amino acid transporters; GHRKO, growth hormone receptor knockout; GluCEST, glutamate chemical exchange saturation transfer; iGluR, ionotropic glutamate receptors; MR, magnetic resonance; MRS, magnetic resonance spectroscopy; mGluR, metabotropic glutamate receptors; MCI, mild cognitive impairment; NMDAr, N-methyl-D-aspartate receptors; PET, positron emission tomography; VGLUT, vesicular glutamate transporter.
